# Estimation of short-course systemic corticosteroid risks on adverse outcomes in childhood asthma

**DOI:** 10.1186/s13223-026-01018-0

**Published:** 2026-02-24

**Authors:** Brian R. Earl, Ewa Sucha, Richard Webster, Alexandra Ahmet, Dhenuka Radhakrishnan

**Affiliations:** 1 Department of Paediatrics, Children’s Hospital of Eastern Ontario (CHEO), 401 Smyth Road, Ottawa, ON K1H 8L1 Canada; 2 CHEO Research Institute, Ottawa, ON Canada; 3 Department of Pediatrics, University of Ottawa, Ottawa, ON Canada

**Keywords:** Pediatrics, Asthma exacerbations, Corticosteroids, Steroid bursts, Adverse outcomes, Emergency department

## Abstract

**Background and objectives:**

Short-course systemic corticosteroids (SCS) are recommended for moderate-to-severe pediatric asthma exacerbations, though frequent courses may cause adverse outcomes. We examined the risk of adverse outcomes in asthmatic children who received multiple SCS courses for exacerbation management.

**Methods:**

We conducted a retrospective study of children aged 1–16 years with an asthma emergency department presentation/hospitalization between October 1, 2017, and February 28, 2021. Using a Prentice-Williams-Peterson total time model, we compared steroid-associated adverse outcomes among children who received or did not receive short courses of SCS for asthma exacerbations over ≥ 24-months.

**Results:**

Among 2009 eligible children, 1468 received ≥ 1 SCS course for asthma exacerbations and 541 did not receive SCS. Overall, there was no increase in the risk of recurrent SCS-associated adverse outcomes in those exposed to SCS (aHR = 0.95, 95% CI 0.74–1.23, *p* = 0.7), however, the number of SCS courses received significantly affected the risk of recurrent adverse outcomes (*p* = 0.029). Children receiving 2 SCS courses had a reduced risk of adverse outcomes (aHR = 0.49, 95% CI: 0.28–0.88), while those receiving ≥ 4 SCS courses experienced a non-significant, yet clinically meaningful elevated risk of adverse outcomes (aHR = 2.30, 95% CI 0.92–5.80).

**Conclusion:**

This study shows that SCS administered for pediatric asthma exacerbations are generally safe; however, complication risk may be increased when receiving 4 + SCS courses and synergistic with higher-dose inhaled corticosteroids.

**Supplementary Information:**

The online version contains supplementary material available at 10.1186/s13223-026-01018-0.

## Introduction

Uncontrolled asthma in children impacts quality of life, and asthma-related costs place an unacceptable burden on healthcare systems [1]. In Canada, only 11% of children achieve adequate asthma control [2], translating to high rates of emergency department (ED) care, where ~ 35% of these children receive systemic corticosteroid (SCS) therapy [3]. The Global Initiative for Asthma and Canadian Thoracic Society guidelines suggest the early administration of SCSs in all but the mildest exacerbations, to reduce hospital and ED readmissions [4–6]. In geographically disparate cohorts of asthmatic children, rates of annual SCS prescriptions reach almost 50% along with very high rates of repeat SCS prescriptions [7–9]. Despite their demonstrated efficacy and perceived safety, studies involving two large adult cohorts identified that those receiving a single SCS prescription were at greater risk of multiple acute complications [10, 11]. Moreover, adults receiving ≥ 4 annual short-course SCS prescriptions for asthma exacerbations, developed chronic complications including osteoporosis, hypertension, obesity, and cataracts [12]. In asthmatic children, exposure to periodic SCS may impart a higher risk of long-term complications [13]. A population-based pediatric study demonstrated an association between SCS bursts and GI bleeding, sepsis, and pneumonia [14]. Multiple SCS bursts are also correlated with a dose-dependent reduction in bone mineral accretion in asthmatic children [15–17]. In all studies, follow-up did not exceed a few months. More recent evidence from a large, representative American cohort has indicated that children receiving a minimum of 1–3 annual short courses of SCS for acute asthma are at greater risk of SCS-related complications [18].

While an association between multiple SCS bursts and complications amongst children is emerging, uncertainty exists due to limited studies, short follow-up and a lack of reporting [19, 20]. This relationship has not been studied within a population of children in a publicly funded, universal access healthcare system as available in Canada. Therefore, we conducted a retrospective cohort study with long-term follow up to explore the effect of multiple SCS bursts on well-documented SCS-associated consequences (i.e., gastrointestinal, infectious, endocrine, or ocular adverse outcomes), amongst asthmatic children who receive acute care at a Canadian tertiary care pediatrics hospital. We hypothesized that children receiving frequent SCS bursts would have a greater risk for adverse outcomes. Broadening our understanding of the complications associated with short courses of SCS in asthmatic children will facilitate more personalized treatment protocols and patient monitoring.

## Methods

### Study design and setting

This is a single-center, retrospective comparative cohort study of children between 1 and 16 years seen in the ED or hospitalized for a diagnosis of asthma and treated with or without SCS at the Children’s Hospital of Eastern Ontario (CHEO). CHEO is a tertiary-level pediatric hospital with a catchment area of 2 million people, and 75,000 annual visits to the ED. This study was approved by the CHEO Research Ethics Board and is reported in accordance with the RECORD statement [21].

### Data sources

For all patient encounters from October 2017 onwards, we used clinical data from the CHEO Epic electronic medical record (EMR). Information on medical diagnoses from ED, inpatient and outpatient visits were defined using International Statistical Classification of Diseases and Related Health Problems, 10th Revision (ICD-10) diagnostic codes. Data on medication prescriptions were identified using their unique Drug Identification Number. Locally collected health administrative data available through the CHEO Data Warehouse were used to define medical diagnoses and healthcare visits prior to October 2017. Using these data sources, we identified all children with an ED visit for asthma during the study accrual period and collected information on age, sex, details of subsequent ED visits or hospitalizations, and number of SCS courses administered (See Supplementary Table 1 for definitions of SCS course, Additional File 1). All relevant comorbid diagnoses and adverse outcomes were identified using ICD-10 codes (See Supplementary Tables 2, and 3, Additional File 1). Asthma severity at triage was ascertained using the pediatric respiratory assessment measure (PRAM) score, and/or the Canadian triage acuity score (CTAS). A PRAM score > 3 represents moderate-to-severe exacerbations and is associated with SCS administrations during ED asthma encounters [22, 23]. Similarly, a CTAS score 1–3 indicates urgent or emergent symptoms and corresponds to a moderate-to-severe exacerbation [24] (See Supplementary Table 1, Additional File 1).

### Study population

**Fig. 1:**
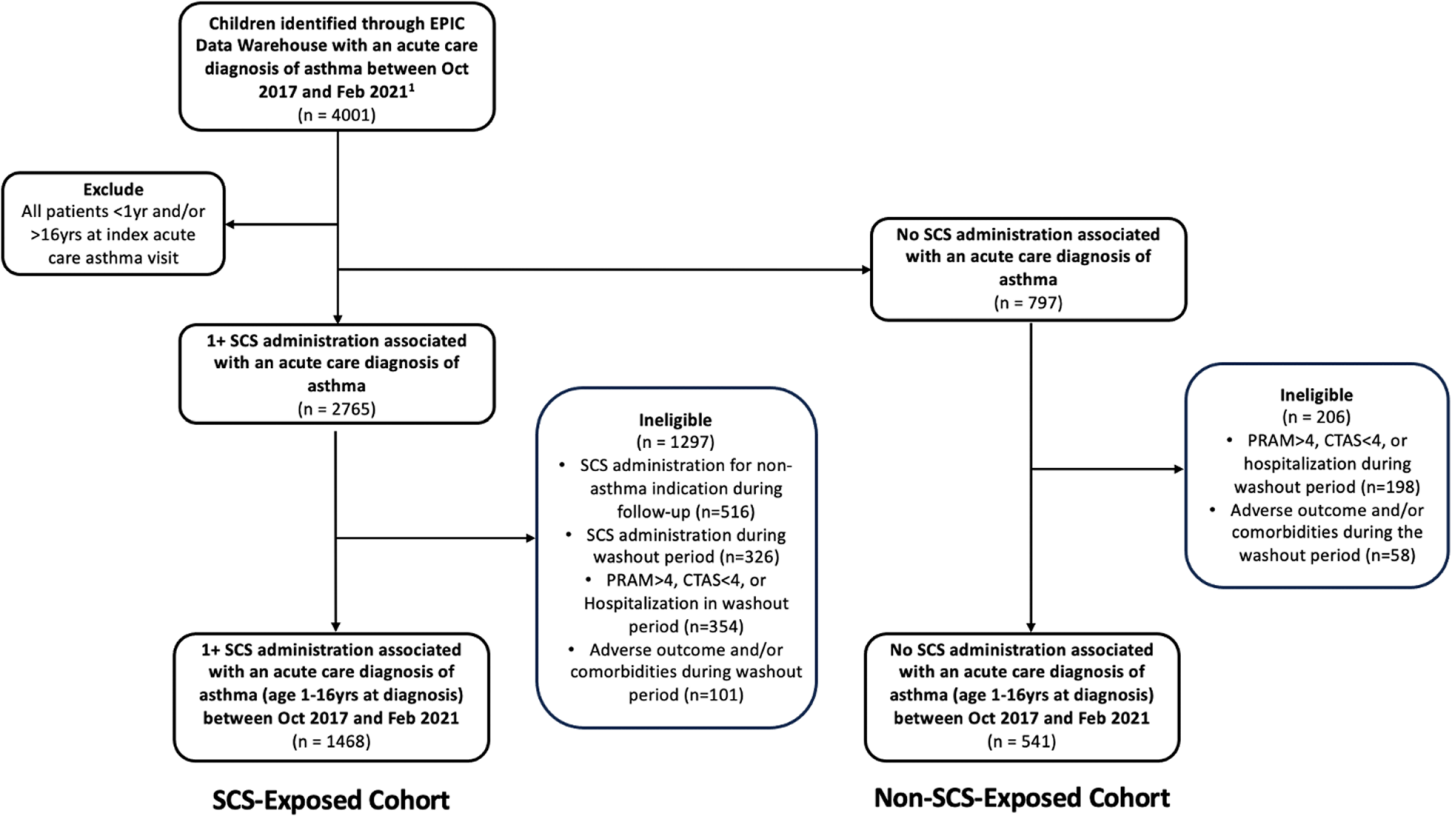
Study cohort selection. Abbreviation: CTAS = Canadian triage acuity score, PRAM = pediatric respiratory assessment measure, SCS = systemic corticosteroids. Patients without Ontario postal code, ≥24 months of consecutive follow-up data, or ≥3 years of consecutive washout period data were already excluded from initial starting population

We included all children aged ≥ 1 to ≤ 16 years with a primary diagnosis of asthma (using ICD codes J45/46) during an ED encounter or hospital admission, between October 2017 and February 2021. This timeframe was chosen to coincide with the availability of Epic EMR data, which is more detailed than health administrative data. Our study duration spanned October 21, 2014 – April 30, 2023, which enabled a minimum 3-year washout period and a minimum 24-month follow-up period. Children < 1 year were excluded due to challenges distinguishing a diagnosis of asthma from bronchiolitis (Fig. 1).

### Cohort development

Participants in the SCS-exposed cohort (cases) were assigned an index date for their first asthma ED visit during the accrual period where they received SCS. Subsequent asthma ED/inpatient encounters over ≥ 24 months with a corresponding SCS prescription were captured. Cases who received any SCS prescriptions for a primary diagnosis other than asthma, were excluded.

A non-SCS-exposed cohort (controls) consisted of participants with an asthma ED visit during the accrual period where no SCS was administered. This cohort required a follow-up observation of ≥ 24 months, with no SCS prescriptions.

Any SCS-associated adverse outcomes during follow-up were captured for both cohorts using ICD-10 codes (See Supplementary Table 3, Additional File 1). Cases and controls were excluded if diagnosed with any comorbid conditions expected to require SCS treatment (See Supplementary Table 2, Additional File 1).

### Washout period

Both cases and controls were observed from the time of birth, or for a minimum of 3 years prior to their index date to ensure a minimal likelihood of previous SCS treatment. Prior to 2017, comprehensive prescribing data was not available, so we developed a composite measure to detect likely SCS prescriptions that consisted of: available prescribing data, asthma severity during ED/Inpatient encounters (i.e., CTAS < 4 or PRAM > 3), and/or hospitalization. Participants with any comorbidities expected to require SCS therapy or diagnosed with any SCS-associated adverse outcomes during the washout period were also excluded. While fractures and pneumonia were adverse outcomes recorded in this study, children with these conditions during the washout were not excluded given their high frequency in children with asthma, and because pneumonia can be related to poor asthma control.

### Covariates

Covariates hypothesized to confound the association of SCS and adverse events were captured at baseline (Table 1).

**Table 1:** Baseline characteristics and systemic corticosteroid exposure

	SCS-Exposed Cohort (N = 1468)	Non-SCS-Exposed Cohort (N = 541)
*Age at Index (y)*		
Median (IQR)	3.0 (1.0, 6.0)	7.0 (4.0, 11.0)
*Age groups at Index*		
1–5 years old, n (%)	1056 (71.9%)	191 (35.3%)
6–16 years old, n (%)	412 (28.1%)	350 (64.7%)
*Sex*		
Female, n (%)	530 (36.2%)	245 (45.5%)
*First PRAM at index date*		
Mean (SD)	6.8 (2.6)	1.5 (1.8)
*Follow-up (y)*		
Median (IQR)	4.0 (3.3, 4.7)	4.2 (3.3, 5.0)
*Washout (y)*		
Median (IQR)	3.0 (1.0, 3.0)	3.0 (2.0, 3.0)
# of SCS courses, n (%)		
0	0 (0%)	541 (100%)
1	1113 (75.8%)	0 (0%)
2	224 (15.3%)	0 (0%)
3	71 (4.8%)	0 (0%)
4 +	60 (4.1%)	0 (0%)
*Deprivation Quintiles, n (%)*		
1	599 (41.3%)	229 (42.7%)
2	274 (18.9%)	97 (18.1%)
3	196 (13.5%)	73 (13.6%)
4	157 (10.8%)	55 (10.3%)
5	225 (15.5%)	82 (15.3%)

IQR = interquartile range, PRAM = pediatric respiratory assessment measure, SCS = systemic corticosteroids, SD = standard deviation

Socioeconomic status was approximated using the neighbourhood material deprivation quintile, which is a dimension of the Ontario Marginalization (ON-Marg) Index [25] and was linked via postal code. ON-Marg is a validated index (where 1 = least marginalized and 5 = most marginalized) and is associated with negative health outcomes, including asthma [26].

### Outcomes

The primary outcome was the development of any SCS-associated adverse outcomes during the follow-up period, as identified using ICD-10 codes (See Supplementary Table 3, Additional File 1).

### Statistical analysis

Baseline characteristics were compared between cohorts using frequency counts, percentages and Fisher’s Exact Test for categorical variables as well as means, medians, standard deviations, and t-tests for continuous variables. A 2-tailed *P* < 0.05 was considered statistically significant.

To determine an association between cumulative SCS bursts and the development of steroid-associated complications, we utilized the Cochrane Armitage test for trend in unadjusted data, and the Prentice-Williams-Peterson (PWP) model with a total time scale measured from the beginning of the study (TT), adjusted for patient age and sex. To fit the PWP-TT, the dataset was truncated after a patient experienced a fourth event (i.e., all adverse events after the fourth per patient were discarded). This cut-off was required to obtain stable estimates. Dependence of recurrent events within the same subject was accounted for using robust standard errors. Results were expressed as adjusted relative rates (RR) and 95% Wald confidence intervals (CI).

### Sensitivity analyses

Analyses were replicated to exclude those patients living outside Ottawa to increase the likelihood that patients enrolled in the study received acute care services at CHEO. An unpaired two-samples Wilcoxon test was used to assess whether those patients with a postal code within Ottawa had more CHEO hospital encounters compared to those residing outside Ottawa.

To account for potential confounding effects from inhaled corticosteroid (ICS) exposure, analyses were repeated after excluding those patients followed in the CHEO respirology clinic, which provides care for children with moderate-to-severe asthma who are treated with moderate-high dose ICS therapy.

To account for our exclusion of patients with croup from our primary analysis, which may have inadvertently eliminated asthmatic patients, we performed a sensitivity analysis with re-inclusion of children with croup.

Finally, we repeated all analyses with exclusion of pneumonia as an outcome, given its anticipated high frequency in the cohort and it also being an indicator of poor asthma control.

## Results

Between October 1, 2017, and February 28, 2021, 4001 children aged 1–16 years old presented to the CHEO ED with asthma, and 2765 received an SCS during the same visit. Following application of inclusion/exclusion criteria, the final study cohort included 1468 in the SCS-exposed cohort and 541 in the SCS-non-exposed cohort (Fig. 1). Notable differences in baseline characteristics (Table 1) included a significantly younger age at index (3.0$;$ 1.0–6.0 vs 7.0; 4.0–11.0, *p* < 0.001), an over-representation of pre-school asthma (71.9%), and significantly fewer females (36.2%) in the SCS-exposed cohort. Most SCS-exposed cases (75.8%) received a single SCS course during the follow-up window. In unadjusted analysis, there was no difference in the total frequency of adverse outcomes between the SCS exposed and non-exposed cohorts (19.6% vs 16.3%, *p* = 0.094, See Supplementary Table 4, Additional File 1), though bacterial pneumonia was more frequent in the SCS-exposed cohort (12.1% vs 4.3%, *p* < 0.001). We did observe a significant trend towards an increase in adverse outcomes in the SCS-exposed cohort as the number of SCS courses increased (*p* = 0.01, Supplementary Table 5, Additional File 1).

After adjustment for age, sex and degree of marginalization, there was no significant association between SCS exposure and recurrent adverse outcomes (aHR = 0.95, 95% CI 0.74–1.23, *p* = 0.7, Table 2).

**Table 2:** Risk of recurrent adverse outcomes in children exposed versus unexposed to systemic corticosteroids for asthma

	Adjusted		
Characteristic	aHR	95% CI	*p*-value
*Cohort*			
Non-SCS-Exposed	–	–	
SCS-Exposed	0.95	0.74, 1.23	0.71
Age at Index	1.00	0.98, 1.03	0.73
Sex			0.63
Female	—	–	
Male	0.95	0.77, 1.17	
Deprivation quintiles			0.070
1	–	–	
2	1.06	0.79, 1.42	
3	1.24	0.94, 1.64	
4	0.72	0.44, 1.15	
5	0.81	0.61, 1.07	

CI = confidence interval, aHR = hazard ratio, SCS = systemic corticosteroids

When the recurrent event analysis was adjusted for the number of SCS courses received, a dose-associated effect of SCS courses on recurrent adverse outcomes was observed (overall *p* = 0.029, Table 3). Children receiving 2 SCS courses had a reduced risk of adverse outcomes (aHR = 0.49, 95% CI 0.28–0.88, Table 3) compared to children receiving a single course. For children receiving ≥ 4 SCS courses there was a non-significant, yet clinically meaningful increase in the risk of adverse outcomes (aHR = 2.30, 95% CI 0.92–5.80, Table 3).

**Table 3:** Risk of recurrent adverse outcomes in children by systemic corticosteroid courses for asthma

	Adjusted		
Characteristic	aHR	95% CI	*p*-value
Number of SCS Courses			0.029
0	–	–	
1	0.99	0.77, 1.27	
2	0.49	0.28, 0.88	
3	0.71	0.27, 1.90	
4 or more	2.30	0.92, 5.80	
Age at Index	1.00	0.98, 1.03	0.93
Sex			0.60
Female	–	–	
Male	0.94	0.76, 1.17	
Deprivation quintiles			0.084
1	–	–	
2	1.05	0.78, 1.40	
3	1.23	0.93, 1.63	
4	0.72	0.45, 1.15	
5	0.81	0.60, 1.07	

CI = confidence interval, aHR = hazard ratio,

SCS = systemic corticosteroids

After excluding respirology clinic-managed patients from this analysis, the dose-association was no longer significant (overall *p* = 0.29, Table 4) and the previously seen apparent increase in recurrent adverse events at ≥ 4 SCS courses diminished (aHR = 1.38, 95% CI 0.35–5.39, Table 4).

**Table 4:** Recurrent adverse outcome risk in children by systemic corticosteroid courses for asthma, without respirology clinic assessment

	Adjusted		
Characteristic	aHR	95% CI	*p*-value
Number of SCS Courses			0.29
0	–	—	
1	1.16	0.85, 1.59	
2	0.56	0.25, 1.23	
3	0.89	0.28, 2.76	
4 or more	1.38	0.35, 5.39	
Age at Index	1.01	0.98, 1.04	0.49
Sex			0.93
Female	–	–	
Male	1.01	0.78, 1.30	
Deprivation quintiles			0.36
1	–	–	
2	1.11	0.80, 1.53	
3	1.00	0.72, 1.40	
4	0.71	0.40, 1.24	
5	0.78	0.54, 1.12	

Abbreviation: CI = confidence interval, aHR = adjusted hazard ratio, SCS = systemic corticosteroids

In a second sensitivity analysis with bacterial pneumonia excluded as an adverse outcome, again, there was no longer a significant dose association between SCS exposure and an increased risk of recurrent adverse events (overall *p* = 0.31, Table 5). Moreover, no significant association was observed with 2 SCS courses (aHR = 0.46, 95% CI 0.17–1.25, Table 5), and there was clearly no association at ≥ 4 SCS courses (aHR = 2.23, 95% CI 0.62, 7.20, Table 5).

**Table 5:** Recurrent adverse outcome risk by systemic corticosteroid courses for childhood asthma, excluding pneumonia

	Adjusted		
Characteristic	aHR	95% CI	*p*-value
Number of SCS Courses			0.31
0	–	–	
1	1.00	0.73, 1.37	
2	0.46	0.17, 1.25	
3	0.60	0.14, 2.52	
4 or more	2.23	0.62, 7.95	
Age at Index	1.02	0.99, 1.05	0.29
Sex			0.43
Female	–	–	
Male	0.89	0.68, 1.18	
Deprivation quintiles			0.16
1	–	–	
2	1.29	0.88, 1.88	
3	1.36	0.95, 1.96	
4	0.78	0.43, 1.41	
5	0.88	0.59, 1.32	

CI = confidence interval, aHR = adjusted hazard ratio, SCS = systemic corticosteroids

Re-including patients with comorbid croup diagnoses within our study sample (n = 2074), did not change the results of any analyses (See Supplementary Table 6, Additional File 1). Additional analysis to exclude patients living outside Ottawa was not pursued as no significant difference in frequency distributions of CHEO hospital encounters for those living within and outside Ottawa was observed (See Supplementary Fig. 1, Additional File 1).

## Discussion

This study evaluated steroid-associated adverse outcomes in a large cohort of children who received short courses of SCS during ED visits or hospitalizations for asthma at a tertiary pediatric centre. We found that SCS for pediatric asthma exacerbations are generally safe with no globally increased risk of recurrent adverse events. However, we did note a clinically meaningful, but not statistically significant increase in the risk of recurrent adverse events among children receiving ≥ 4 SCS courses. This suggests a potential threshold effect after exposure to a higher number of SCS courses, which was not independently significant in our study, likely due to the very small proportion of our cohort exposed to frequent SCS courses. These observations align with previous studies, including that of Sullivan et al*.* who also found that children exposed to ≥ 4 SCS annual courses had a 2.9 × increased odds of complications [10, 14, 18, 27].

Amongst all patients receiving SCS courses in this study, pneumonia was the most prevalent adverse outcome, which is also known to inversely correlate with asthma control [28]. In our sensitivity analysis where pneumonia was removed as an adverse outcome, the dose-association in recurrent adverse events was no longer present, particularly the unexpected finding of a reduction in the recurrent risk of adverse events among those receiving 2 SCS courses. A similar pattern emerged in our sensitivity analysis where patients followed in our centre’s respirology clinic were removed. This may suggest that receiving 2 SCS courses (i.e., reflecting 2 severe exacerbations) triggered more focus on preventative interventions to improve asthma control in the respirology clinic (e.g., asthma education, improving adherence), resulting in a reduction in the frequency of pneumonias. Excluding pneumonia also negated the previously noted dose-associated risk of adverse outcomes in those receiving ≥ 4 SCS courses. Thus, in our study, whether the risk of pneumonia reflects an SCS-associated adverse event or is just a reflection of asthma control is in question. Despite this, in a large population-based pediatric study of single SCS exposures for various indications, an increased risk of pneumonia was identified [14], suggesting a correlation between short courses of SCS and the development of pneumonia irrespective of the presence of asthma.

Our study also identified fewer SCS complications compared to others, potentially related to our stricter definition of adverse outcomes. Sullivan et al*.* reported on a composite adverse outcome that included milder and transient complications such as vomiting, and behavioural disturbances. It is known that these types of mild SCS-associated adverse reactions, are treatment-limited [19, 20], and were not the focus of the present study. We also did not detect an increased risk for serious SCS complications such as sepsis, GI bleeding, osteoporosis and adrenal insufficiency. This differs from the results of Yao et al. [14] who observed the development of sepsis and GI bleeding after a single SCS course. This may be explained by the large numbers needed to harm in the aforementioned study [14] and that SCS courses up to 14 days were considered, compared to our study where a course was defined as ≤ 5 days, consistent with pediatric asthma exacerbation management standards. This longer duration of exposure may have contributed to the increased risk of adverse events observed in this prior study, given corticosteroid complications are dose and time-related [29, 30].

Patients in our study were exposed to fewer SCS courses than cohorts from comparable studies. Most patients in our cohort received a single SCS course, with only ~ 4% receiving ≥ 4 SCS courses. This may be because our study exclusively investigated patients at a tertiary pediatrics centre, compared to broader community-based asthma cohorts in other studies [18]. Moreover, guideline-directed asthma care is greater in pediatric EDs [31], along with severity-based treatment algorithms to ensure judicious SCS prescribing. Similarly, prescription of ICS at ED discharge is routine at our institution, and this reduces asthma ED relapses [32–34], but is not standard practice in many EDs [35, 36]. The lower burden of SCS exposure in our study likely contributed to the fewer number of SCS-associated complications observed. Moreover, pre-school wheezing is known to be highly heterogenous, with a population of children remitting without persistence of asthma symptoms [37]. Given children as young as 1-year-old were identified in this study, it is possible that a proportion of these children with an ED or inpatient visit diagnosis for asthma, may have instead experienced a transient episode of virally induced wheeze. These children would be expected to have fewer subsequent asthma-like presentations, potentially leading to fewer SCS courses and SCS-associated adverse events. Similarly, these children are likely to have less chronic disease burden and may be at lower risk for developing this study’s adverse outcomes of interest.

There is growing concern about the synergistic effects of high-dose ICS and intermittent SCS exposure [38]. While prior literature has argued that oral steroid exposure in asthma contributes more significantly to adverse outcomes [38, 39], recent data have identified the potential dose-dependent adverse effects of ICS, including adrenal suppression [40, 41]. We attempted to control for this by removing patients likely receiving higher doses of ICS (i.e., those with more severe asthma followed at our centre’s respirology clinic) from our analysis. This was associated with negating the dose-associated increased risk of adverse events, possibly suggesting that side effects of intermittent SCS courses may be augmented by higher ICS doses, though our study design does not permit full exploration of this question. In fact, this study may not have captured certain important adverse events (i.e., adrenal suppression) among children on higher ICS doses, if they had received outpatient OCS or did not have an asthma ED visit during the study window, and thus we continue to advocate for screening of this type of complication in this population. Moreover, we were unable to assess ICS adherence and while we attempted to control for those likely receiving higher ICS doses, we cannot comment on the consistency of their administration.

## Study limitations

In this study we were unable to adjust for baseline asthma severity due to lack of detailed ICS dosing and adherence data or lung function in our datasets. Propensity score matching by asthma severity or likelihood to receive SCS was considered, however, after excluding participants potentially exposed to SCS for non-asthma indications and/or those who developed SCS-related adverse outcomes during the washout period, there were few remaining factors to include in a propensity score. Accordingly, we adjusted for remaining variables (i.e., age, sex, and marginalization) and used sensitivity analyses to better characterize our results.

Data capture in this study was restricted to records available for SCS prescribing at CHEO. Therefore, any potential SCS prescriptions from surrounding facilities were unaccounted for, potentially underestimating the true annual exposure of SCS, while also potentially leading to the unintended inclusion of children that have received SCS for non-asthma indications. Specific durations of SCS prescriptions were not controlled for in this study, however, an SCS course was limited to ≤ 3 days of dexamethasone or ≤ 5 days of prednisone, which are both the typical maximal durations as per pediatric asthma standard of care. Moreover, given this study’s retrospective design, information about adherence to SCS courses could not be obtained. Adverse outcomes may also be underreported if care was sought at another facility. We suspect this would be true for routine conditions (i.e., pneumonia, viral illness, etc.) and less relevant for conditions requiring subspeciality involvement (i.e., osteoporosis, fractures, etc.) that is only provided at CHEO for children in our region. Moreover, by analyzing the frequency of CHEO encounters by region, we found it was equivalent amongst those patients living both outside and within the Ottawa region.

## Conclusions

In this study, we demonstrated that intermittent SCS courses for the management of asthma exacerbations at a Canadian tertiary pediatrics centre are generally safe. Regardless, we recommend caution beyond ≥ 4 SCS courses, especially with concomitant use of moderate-high dose ICS, as this may represent a threshold for increased risk of adverse outcomes. Ongoing inquiry to differentiate the influence of higher doses of ICS and short-course SCS on complications in children with asthma represents an important domain of research. Similarly, we recommend exercising SCS stewardship in childhood asthma and exploring the efficacy of lower dose and shorter duration SCS courses for acute asthma exacerbation management. This study emphasizes the importance of achieving good asthma control as an approach to minimize the need for repetitive SCS courses in pediatric asthma.

## Supplementary Information

Below is the link to the electronic supplementary material.

Supplementary Material 1

## Abbreviations

CHEO: Children’s hospital of eastern Ontario
CTAS: Canadian triage acuity score
ED: Emergency department
EMR: Electronic medical record
ICD-10: International statistical classification of diseases and related health problems
10th Revision, ICS: Inhaled corticosteroids
PRAM: Pediatric respiratory assessment measure
SCS: Systemic corticosteroids

## Declarations

### Data availability

The datasets used and/or analysed during the current study are available from the corresponding author on reasonable request.

## References

[1] Masoli M, Fabian D, Holt S, Beasley R. Global initiative for asthma (GINA) program. the global burden of asthma: executive summary of the GINA dissemination committee report. Allergy. 2004;59(5):469–78. 10.1111/j.1398-9995.2004.00526.x. 15080825 10.1111/j.1398-9995.2004.00526.x

[2] Cope SF, Ungar WJ, Glazier RH. Socioeconomic factors and asthma control in children. Pediatr Pulmonol. 2008;43(8):745–52. 10.1002/ppul.20817. 18615669 10.1002/ppul.20847 PMC4940183

[3] Lougheed MD, Garvey N, Chapman KR, Cicutto L, Dales R, Gay AG, et al. Variations and gaps in management of acute asthma in Ontario emergency departments. Chest. 2009;135(3):724–36. 10.1378/chest.08-1039. 19017869 10.1378/chest.08-0371

[4] Wei J, Lu Y, Han F, Zhang J, Liu L, Chen Q. Oral dexamethasone vs oral prednisone for children with acute asthma exacerbations: a systematic review and meta-analysis. Front Pediatr. 2019;7:503. 10.3389/fped.2019.00503. 31921718 10.3389/fped.2019.00503 PMC6923200

[5] Bhogal SK, McGillivray D, Bourbeau J, Benedetti A, Bartlett S, Ducharme FM. Early administration of systemic corticosteroids reduces hospital admission rates for children with moderate and severe asthma exacerbation. Ann Emerg Med. 2012;60(1):84-91.e3. 10.1016/j.annemergmed.2012.02.019. 22410507 10.1016/j.annemergmed.2011.12.027

[6] Ortiz-Alvarez O, Mikrogianakis A. Managing the paediatric patient with an acute asthma exacerbation. Paediatr Child Health. 2012;17(5):251–5. 10.1093/pch/17.5.251. 23633900 10.1093/pch/17.5.251 PMC3381918

[7] Farber HJ, Silveira EA, Vicere DR, Kothari VD, Giardino AP. Oral corticosteroid prescribing for children with asthma in a Medicaid managed care program. Pediatrics. 2017;139(5):e20163489. 10.1542/peds.2016-3489. 10.1542/peds.2016-4146 28557753

[8] Arabkhazaeli A, Vijverberg SJH, van der Ent CK, Raaijmakers JAM, van der Maitland- Zee AH. High incidence of oral corticosteroids prescriptions in children with asthma in early childhood. J Asthma. 2016;53(10):1012–7. 10.3109/02770903.2016.1159292. 27187595 10.1080/02770903.2016.1185439

[9] Mudd K, Bollinger ME, Hsu VD, Donithan M, Butz A. Pharmacy fill patterns in young urban children with persistent asthma. J Asthma. 2006;43(8):597–600. 10.1080/02770900600810652. 17050224 10.1080/02770900600878537

[10] Yao TC, Huang YW, Chang SM, Tsai SY, Wu AC, Tsai HJ. Association between oral corticosteroid bursts and severe adverse events: a nationwide population-based cohort study. Ann Intern Med. 2020;173(5):325–30. 10.7326/M19-3145. 32628532 10.7326/M20-0432

[11] Waljee AK, Rogers MAM, Lin P, Singal AG, Stein JD, Marks RM, et al. Short term use of oral corticosteroids and related harms among adults in the United States: population based cohort study. BMJ. 2017;18(357):j1415. 10.1136/bmj.j1415. 10.1136/bmj.j1415 PMC6284230 28404617

[12] Sullivan PW, Ghushchyan VH, Globe G, Schatz M. Oral corticosteroid exposure and adverse effects in asthmatic patients. J Allergy Clin Immunol. 2018;141(1):110-116.e7. 10.1016/j.jaci.2017.04.038. 28456623 10.1016/j.jaci.2017.04.009

[13] Kapadia CR, Nebesio TD, Myers SE, Willi S, Miller BS, Allen DB, et al. Endocrine effects of inhaled corticosteroids in children. JAMA Pediatr. 2016;170(2):163–70. 10.1001/jamapediatrics.2015.3344. 26720105 10.1001/jamapediatrics.2015.3526

[14] Yao TC, Wang JY, Chang SM, Chang YC, Tsai YF, Wu AC, et al. Association of oral corticosteroid bursts with severe adverse events in children. JAMA Pediatr. 2021;175(7):723–9. 10.1001/jamapediatrics.2021.0736. 33871562 10.1001/jamapediatrics.2021.0433 PMC8056312

[15] Kelly HW, Van Natta ML, Covar RA, Tonascia J, Green RP, Strunk RC. Effect of long-term corticosteroid use on bone mineral density in children: a prospective longitudinal assessment in the childhood asthma management program (CAMP) study. Pediatrics. 2008;122(1):e1–8. 10.1542/peds.2007-3754. 18595975 10.1542/peds.2007-3381 PMC2928657

[16] Ducharme FM, Chabot G, Polychronakos C, Glorieux F, Mazer B. Safety profile of frequent short courses of oral glucocorticoids in acute pediatric asthma: impact on bone metabolism, bone density, and adrenal function. Pediatrics. 2003;111(2):376–83. 10.1542/peds.111.2.376. 12563067 10.1542/peds.111.2.376

[17] Harris M, Hauser S, Nguyen TV, Kelly PJ, Rodda C, Morton J, et al. Bone mineral density in prepubertal asthmatics receiving corticosteroid treatment. J Paediatr Child Health. 2001;37(1):67–71. 10.1046/j.1440-1754.2001.00462.x. 11168874 10.1046/j.1440-1754.2001.00628.x

[18] Sullivan PW, Ghushchyan VH, Skoner DP, Park S, Zeiger RS. Complications and health care resource utilization associated with systemic corticosteroids in children and adolescents with persistent asthma. J Allergy Clin Immunol Pract. 2021;9(4):1541-1551.e9. 10.1016/j.jaip.2020.11.022. 33290914 10.1016/j.jaip.2020.11.049 PMC8393544

[19] Fernandes RM, Wingert A, Vandermeer B, Featherstone R, Ali S, Plint AC, et al. Safety of corticosteroids in young children with acute respiratory conditions: a systematic review and meta-analysis. BMJ Open. 2019;9(8):e027394. 10.1136/bmjopen-2018-027394. 10.1136/bmjopen-2018-028511 PMC6688746 31375615

[20] Aljebab F, Choonara I, Conroy S. Systematic review of the toxicity of short-course oral corticosteroids in children. Arch Dis Child. 2016;101(4):365–70. 10.1136/archdischild-2015-309522. 26768830 10.1136/archdischild-2015-309522 PMC4819633

[21] Benchimol EI, Smeeth L, Guttmann A, Harron K, Moher D, Petersen I, et al. The REporting of studies conducted using observational routinely-collected health data (RECORD) statement. PLoS Med. 2015;12(10):e1001885. 10.1371/journal.pmed.1001885. 26440803 10.1371/journal.pmed.1001885 PMC4595218

[22] Chalut DS, Ducharme FM, Davis GM. The preschool respiratory assessment measure (PRAM): a responsive index of acute asthma severity. J Pediatr. 2000;137(6):762–8. 10.1067/mpd.2000.110155. 11113831 10.1067/mpd.2000.110121

[23] Zemek R, Plint AC, Osmond MH, Kovesi T, Correll R, Perri N, et al. Triage nurse initiation of corticosteroids in pediatric asthma is associated with improved emergency department efficiency. Pediatrics. 2012;129(4):671–80. 10.1542/peds.2011-2201. 22430452 10.1542/peds.2011-2347

[24] Gravel J, Gouin S, Goldman RD, Osmond MH, Fitzpatrick E, Boutis K, et al. The Canadian triage and acuity scale for children: a prospective multicenter evaluation. Ann Emerg Med. 2012;60(1):71-7.e3. 10.1016/j.annemergmed.2012.02.027. 22305329 10.1016/j.annemergmed.2011.12.004

[25] Matheson FI, Moloney G, van Ingen T. 2021 Ontario marginalization index: user guide. Toronto: Joint Publication with Public Health Ontario; 2023.

[26] Sheriff F, Agarwal A, Thipse M, Radhakrishnan D. Hot spots for pediatric asthma emergency department visits in Ottawa, Canada. J Asthma. 2022;59(5):880–9. 10.1080/02770903.2021.1910815. 33567912 10.1080/02770903.2021.1887891

[27] Zeiger RS, Sullivan PW, Chung Y, Kreindler JL, Zimmerman NM, Tkacz J. Systemic corticosteroid-related complications and costs in adults with persistent asthma. J Allergy Clin Immunol Pract. 2020;8(10):3455-3465.e13. 10.1016/j.jaip.2020.05.016. 32679349 10.1016/j.jaip.2020.06.055

[28] Castro-Rodriguez JA, Abarca K, Forno E. Asthma and the risk of invasive pneumococcal disease: a meta-analysis. Pediatrics. 2020;145(1):e20191858. 10.1542/peds.2019-1858. 10.1542/peds.2019-1200 PMC6939845 31843863

[29] Prete A, Bancos I. Glucocorticoid induced adrenal insufficiency. BMJ. 2021;374:n1380. 34253540 10.1136/bmj.n1380

[30] Kwon S, Hermayer KL, Hermayer K. Glucocorticoid-induced hyperglycemia. Am J Med Sci. 2013;345(4):274–7. 23531958 10.1097/MAJ.0b013e31828a6a01

[31] Hudgins JD, Neuman MI, Monuteaux MC, Porter J, Nelson KA. Provision of guideline-based pediatric asthma care in US emergency departments. Pediatr Emerg Care. 2021;37(10):507–12. 30624420 10.1097/PEC.0000000000001706

[32] Benito-Fernández J, Onis-González E, Álvarez-Pitti J, Capapé-Zache S, Vázquez-Ronco MA, Mintegi-Raso S. Factors associated with short-term clinical outcomes after acute treatment of asthma in a pediatric emergency department. Pediatr Pulmonol. 2004;38(2):123–8. 15211695 10.1002/ppul.20031

[33] Self TH, Twilla JD, Rogers ML, Rumbak MJ. Inhaled corticosteroids should be initiated before discharge from the emergency department in patients with persistent asthma. J Asthma Allergy. 2009. 10.3109/02770900903274483. 10.3109/02770900903274483 19995133

[34] Adams RJ, Fuhlbrigge A, Finkelstein JA, Lozano P, Livingston JM, Weiss KB, et al. Impact of inhaled antiinflammatory therapy on hospitalization and emergency department visits for children with asthma. Pediatrics. 2001;107(4):706–11. 11335748 10.1542/peds.107.4.706

[35] Schuh S, Zemek R, Plint A, Black KJL, Freedman S, Porter R, et al. Practice patterns in asthma discharge pharmacotherapy in pediatric emergency departments: a pediatric emergency research Canada study. Acad Emerg Med. 2012;19(9):E1019–26. 22978728 10.1111/j.1553-2712.2012.01433.x

[36] Garro AC, Asnis L, Merchant RC, McQuaid EL. Frequency of prescription of inhaled corticosteroids to children with asthma in U.S. emergency departments. Acad Emerg Med. 2011;18(7):767–70. 21762239 10.1111/j.1553-2712.2011.01117.x

[37] Sears M. Predicting asthma outcomes. J Allergy Clin Immunol. 2015;136(4):829–36. 26449797 10.1016/j.jaci.2015.04.048

[38] Bourdin A, Suehs C, Charriot J. Integrating high dose inhaled corticosteroids into oral corticosteroids stewardship. Eur Respir J. 2020. 10.1183/13993003.02193-2019. 31896681 10.1183/13993003.02193-2019

[39] Gray N, Howard A, Zhu J, Feldman LY, To T. Association between inhaled corticosteroid use and bone fracture in children with asthma. JAMA Pediatr. 2018;172(1):57. 29131874 10.1001/jamapediatrics.2017.3579 PMC5833516

[40] Kachroo P, Stewart ID, Kelly RS, Stay M, Mendez K, Dhalin A, et al. Metabolomic profiling reveals extensive adrenal suppression due to inhaled corticosteroid therapy in asthma. Nat Med. 2022;28(4):814–22. 35314841 10.1038/s41591-022-01714-5 PMC9350737

[41] Kwda A, Gldc P, Baui B, Kasr K, Us H, et al. Effect of long term inhaled corticosteroid therapy on adrenal suppression, growth and bone health in children with asthma. BMC Pediatr. 2019;19(1):411. 31684902 10.1186/s12887-019-1760-8 PMC6829958

