# Supplementary material for: Estimation of short-course systemic corticosteroid risks on adverse outcomes in childhood asthma
Source: Allergy Asthma Clin Immunol. 2026 Feb 24;22:14. doi: 10.1186/s13223-026-01018-0 (PMC13037048; doi:10.1186/s13223-026-01018-0)
Supplement: Supplementary file 1 — Supplementary Material 1 [file 13223_2026_1018_MOESM1_ESM.pdf]

Supplementary Figure 1. Distribution of hospital encounters by region of residence.

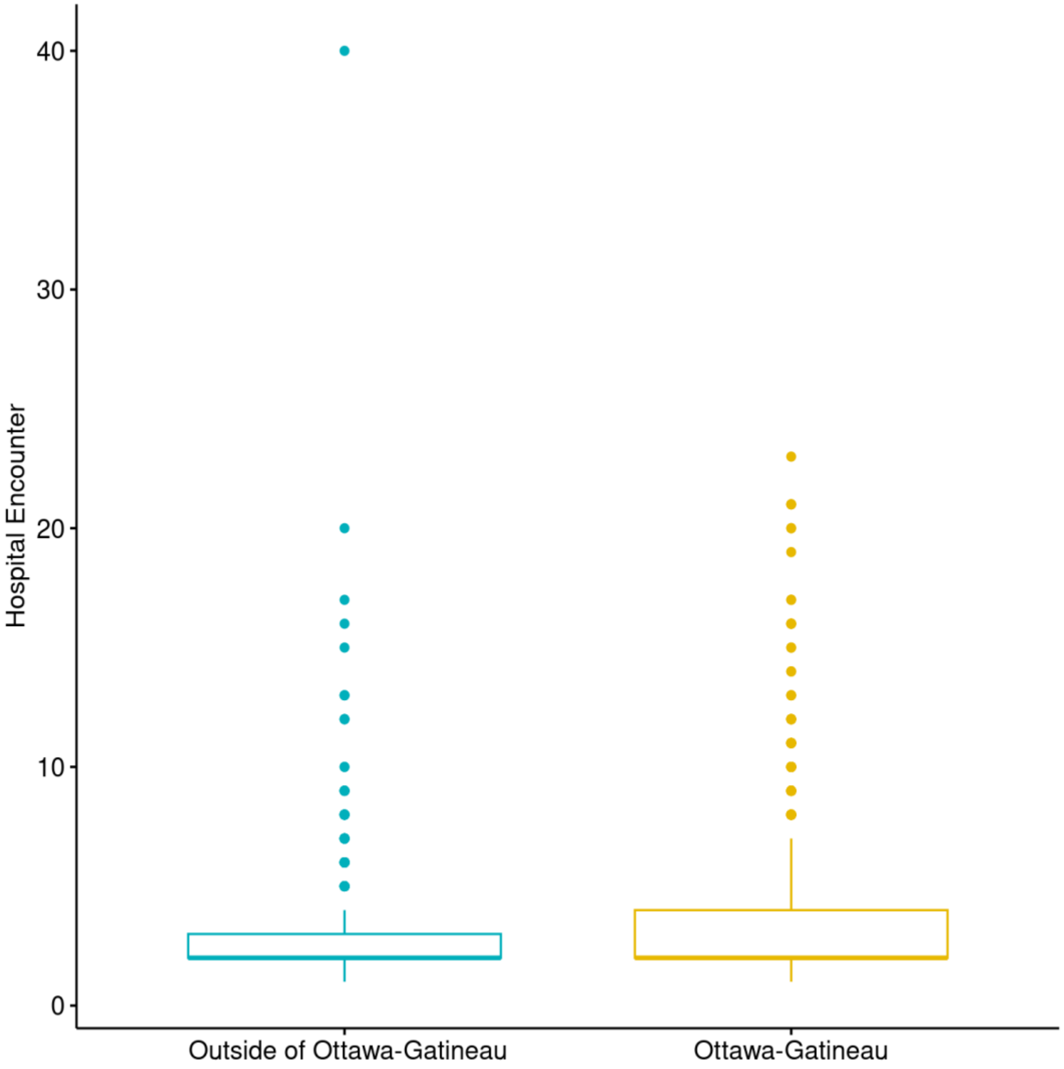

Unpaired Two-Samples Wilcoxon Test was used to assess whether patients residing within the Ottawa region have more hospital encounters than those living outside. *p*-value=0.9938.

Supplementary Table 1. Selection Criteria for Study Population

**Inclusion Criteria**
• Age ≥ 1 and ≤ 16 years at index date
• Children and adolescents with ≥ 1 presentation with a most responsible diagnosis of asthma where an SCS was or was not prescribed<br>○ **SCS Course Definition:**<br>1. PO dexamethasone for ≤ 3 days OR any combination of IV steroids + PO dexamethasone for ≤ 3 days<br>2. PO prednisone for ≤ 5 days OR any combination of IV steroids + PO prednisone for ≤ 5 days
• ≥ 24 months of continuous Epic Data Warehouse data available following index date
• Continuous Epic Data Warehouse data available from birth or ≥ 5 years prior to the index date
**Exclusion Criteria**
• Residents of Quebec (as identified by health insurance provider [i.e. RAMQ])
• Patients with a comorbid illness suspected to require SCS therapy (see Supplemental Table 1)
• Patients with presence of one of this study's adverse outcome conditions during their washout period (see Supplemental Table 2)
• History of suspected SCS use during the washout period, as determined by:<br>○ Available SCS ordering/administration data<br>○ PRAM >3 at asthma ED visit<br>○ CTAS 1-3 at asthma ED visit<br>○ Hospitalization for asthma

Abbreviation: CTAS = Canadian Triage Acuity Score, PRAM = Pediatric Respiratory Assessment Measure, RAMQ = Régie de l'Assurance Maladie du Québec, SCS = Systemic Corticosteroids.

Supplementary Table 2. Comorbid conditions suspected to require systemic corticosteroid Therapy

	Comorbid Diagnoses	ICD-10[1] Codes
**Gastrointestinal Disease**	Crohn's Disease	K50
	Ulcerative Colitis	K51
	Autoimmune Hepatitis	K75.4
	Hypereosinophilic Syndromes	D72.1, K20.0, K52.81
**Rheumatic/Inflammatory Diseases**	Lupus (SLE, Discoid)	M32, L93
	Juvenile Idiopathic Arthritis	M08
	Vasculitides	M30, M31
	Other Systemic Rheumatologic Diseases (Scleroderma, Juvenile Dermatomyositis, Kawasaki Disease, Granulomatosis Polyangiitis, Multifocal Polyangiitis, etc.)	M33-36
	Graft-Versus-Host Disease	D89.81
	Organ Transplant	Z94
	Neuroinflammatory Syndromes	G04, G37.3
**Hem/Onc Diseases**	Malignancy (ALL/AML, Non/Hodgkin's Lymphoma, Osteosarcoma/Ewing's Sarcoma, Neuroblastoma, Wilms' Tumour, CNS/Ocular Tumour)	C00-96, C7A
	Hematologic Diseases (Autoimmune Hemolytic Disease, Langerhans Cell Histiocytosis, etc.)	D59.0, D59.1, D59.8, D59.9
**Respiratory Diseases**	Bronchiectasis	J47
	COPD	J44
	Cystic Fibrosis	E84
	Interstitial Lung Disease	J84.1
**Miscellaneous Disease**	Primary Adrenocortical Insufficiency	E27.1
	Nephrotic/Nephritic Syndrome	N00, N01, N03, N04, N05, N06, N08
	Duchenne Muscular Dystrophy	G71.1

Abbreviation: AML = acute myeloid leukemia, ALL = acute lymphocytic leukemia, COPD = chronic obstructive pulmonary disease, SLE = systemic lupus erythematosus, ICD = International Classification of Disease

Supplementary Table 3. Adverse outcomes suspected to be associated with systemic corticosteroid therapy

	Adverse Conditions	ICD-10[1] Codes
**Gastrointestinal**	GI Ulcers/Bleeding	K25.3, K25.9, K92.2, K27.0-7, K27.9
	Pancreatitis	K85.0-3, K85.8-9
**Infectious**	Sepsis	A41.0-2, A41.4, A41.50-2, A41.58, A41.80, A41.88, A41.9
	Candidiasis	B37.81-83
	Viral Pneumonia	J10-12
	Bacterial Pneumonia	J13-18
**Endocrine**	Hypertension	I10, I15.2, I15.8-9
	Hyperglycemia	R73
	Obesity	E65-66
	Type 2 Diabetes Mellitus	E11
	Dyslipidemia/Hypercholesterolemia	E78
	Metabolic Syndrome	E88.81
	Fracture	S02, S12, S22, S32, S42, S52, S62, S72, S82, S92, M48.4-5, M84, Z87.31, Z87.81
	Decreased Bone Mineral Density	M85.8-9
	Osteoporosis/Osteopenia	M80.0, M80.4-5, M80.8, M80.9, M81.0, M81.4-6, M81.8, M81.9
	Avascular Necrosis	M87.0-1, M87.3, M87.8, M87.9
	Cushing's Syndrome	E24.2, E24.8-9
	Cushingoid Features	L68, L70, L90.6
	Endocrine Short Stature	E34.3
	Adrenal Insufficiency	E27.1, E27.2, E27.3, E27.4
**Ocular**	Cataracts	H26
	Glaucoma	H40

Abbreviation: ICD = International Classification of Disease

Supplementary Table 4. Incidence rates of adverse outcomes in pediatric asthma exacerbations managed with or without systemic corticosteroids.

**Adverse Outcomes**[1]	**SCS-Exposed Cohort** (N=1468)	**Non-SCS-Exposed Cohort** (N=541)	***P*-value**[2]
**Bacterial Pneumonia**			
No	1291 (87.9%)	518 (95.7%)	<0.001
Yes	177 (12.1%)	23 (4.3%)	
**Sepsis**			
No	1468 (100%)	540 (99.8%)	0.27
Yes	0 (0%)	1 (0.2%)	
**Viral Pneumonia**			
No	1439 (98.0%)	534 (98.7%)	0.35
Yes	29 (2.0%)	7 (1.3%)	
**Obesity/Overweight**			
No	1463 (99.7%)	535 (98.9%)	0.079
Yes	5 (0.3%)	6 (1.1%)	
**Dyslipidemia/Hypercholesterolemia**			
No	1467 (99.9%)	541 (100%)	1
Yes	1 (0.1%)	0 (0%)	
**Fracture**			
No	1365 (93.0%)	490 (90.6%)	0.073
Yes	103 (7.0%)	51 (9.4%)	
**Cushingoid Features**			
No	1468 (100%)	540 (99.8%)	0.27
Yes	0 (0%)	1 (0.2%)	
**Endocrine/Short Stature**			
No	1463 (99.7%)	540 (99.8%)	1
Yes	5 (0.3%)	1 (0.2%)	
**Adrenal Insufficiency**			
No	1465 (99.8%)	540 (99.8%)	1
Yes	3 (0.2%)	1 (0.2%)	
**Composite outcome**			
No	1180 (80.4%)	453 (83.7%)	0.094
Yes	288 (19.6%)	88 (16.3%)	

Abbreviation: SCS = systemic corticosteroids

1. Frequencies of incident adverse outcomes throughout study period in SCS-exposed and non-SCS-exposed patients. No events were captured in either cohort for gastrointestinal bleeding, pancreatitis, candidiasis, tuberculosis, hypertension, hyperglycemia, type 2 diabetes mellitus, metabolic syndrome, osteoporosis/osteopenia, avascular necrosis, Cushing's syndrome, cataracts, or glaucoma.
2. Categorical variables were compared using Fisher's Exact Test.

Supplementary Table 5. Proportion of cohort with adverse outcomes stratified by the number of systemic corticosteroid courses received.

	Number of SCS Courses					
	0 (N=541)	1 (N=1113)	2 (N=224)	3 (N=71)	4+ (N=60)	Overall (N=2009)
**Adverse outcomes**						
No	466 (86.1%)	991 (89.0%)	185 (82.6%)	54 (76.1%)	39 (65.0%)	1735 (86.4%)
Yes	75 (13.9%)	122 (11.0%)	39 (17.4%)	17 (23.9%)	21 (35.0%)	274 (13.6%)

Abbreviation: SCS = systemic corticosteroids

Supplementary Table 6. Recurrent adverse outcome risk by systemic corticosteroid courses for childhood asthma, including historical croup diagnoses.

	Adjusted		
**Characteristic**	**aHR**	**95% CI**	***p*-value**
**Number of SCS Courses**			0.025
0	—	—	
1	0.98	0.76, 1.25	
2	0.48	0.27, 0.86	
3	0.69	0.26, 1.83	
4 or more	2.27	0.91, 5.68	
**Age at Index**	1.00	0.98, 1.02	0.909
**Sex**			0.498
Female	—	—	
Male	0.93	0.75, 1.15	
**Deprivation quintiles**			0.136
1	—	—	
2	1.05	0.77, 1.41	
3	1.17	0.89, 1.56	
4	0.75	0.50, 1.13	
5	0.81	0.61, 1.08	

Abbreviation: CI = confidence interval, aHR = adjusted hazard ratio, SCS = systemic corticosteroids
